# Supplementary material for: Visualization of translation termination intermediates trapped by the Apidaecin 137 peptide during RF3-mediated recycling of RF1
Source: Nat Commun. 2018 Aug 3;9:3053. doi: 10.1038/s41467-018-05465-1 (PMC6076264; doi:10.1038/s41467-018-05465-1)
Supplement: Supplementary file 1 — Supplementary Information [file 41467_2018_5465_MOESM1_ESM.pdf]

SUPPLEMENTARY INFORMATION

FOR

Visualization of translation termination intermediates trapped by the Api137 peptide during RF3-mediated recycling of RF1

Michael Graf[1], Paul Huter[1], Cristina Maracci[2], Miroslav Peterek[3], Marina V. Rodnina[2], Daniel N. Wilson[1,4*]

[1] Institute for Biochemistry and Molecular Biology, University of Hamburg, Martin-Luther-King-Platz 6, 20146 Hamburg, Germany

[2] Department of Physical Biochemistry, Max Planck Institute for Biophysical Chemistry, Göttingen, Germany.

[3] Central European Institute of Technology (CEITEC), Masaryk University, Kamenice 5, 62500 Brno, Czech Republic.

[4] Lead Contact

*Correspondence to: daniel.wilson@chemie.uni-hamburg.de

SUPPLEMENTARY FIGURES

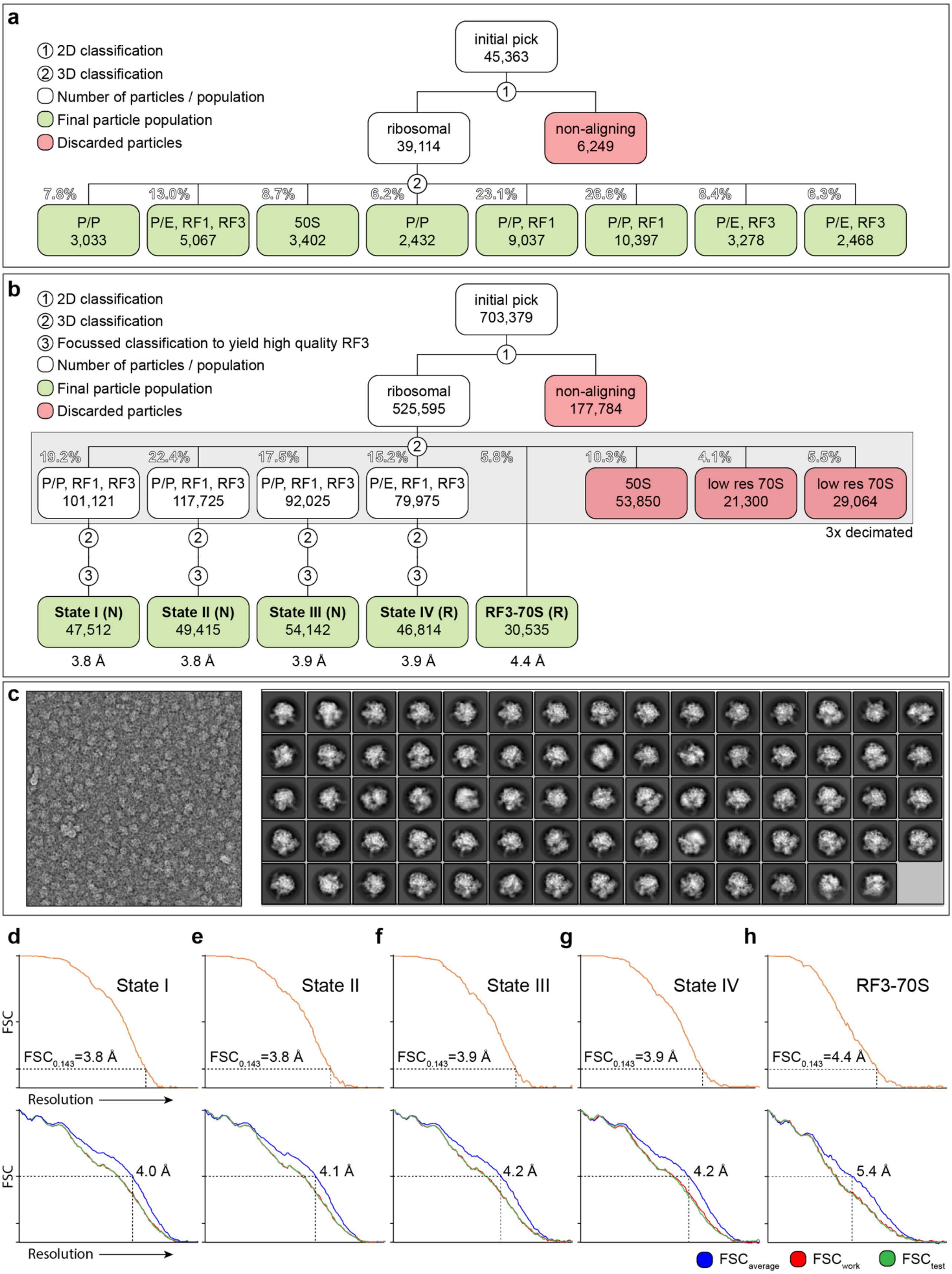

Supplementary Figure 1 Processing of the cryo-EM data of 70S-tRNA-RF1-RF3 complexes. (**a**) Sorting scheme of the cryo-EM data of *in vitro* reconstituted complexes prepared in the absence of API. From a total of 45,363 particles, 6,249 non-aligning particles were discarded after 2D classification. The remaining 39,114 particles were subjected to 3D classification and sorted into 8 different classes. The classes comprise two populations containing only P/P-tRNA, two populations containing P/P-tRNA and RF1, two populations harbouring P/E-tRNA and RF3, one population containing P/E-tRNA, RF1 and RF3, as well as a minor population of 50S subunits. (**b-h**) Processing of 70S-tRNA-RF1-RF3 complexes that were prepared in the presence of API. (**b**) From an initial 703,379 particles, 177,784 non-aligning particles were discarded during 2D classification, yielding 525,595 ribosomal particles. An initial 3D classification was performed sorting the particles into 8 distinct classes, four major populations (states I-IV) containing RF1 and RF3, minor populations containing RF3 only (RF3-70S), vacant 50S subunits or conformational heterogeneous 70S ribosomes. States I to IV were subjected to another round of 3D classification. Moreover, focussed classification was employed to improve density for RF3 in states I-IV. The most stable (sub-)classes were 3D-refined using undecimated particles. (**c**) Example micrograph and 2D class averages. (**d-h)** Fourier Shell Correlation ($FSC_{0.143}$; orange) with the resolution at FSC=0.143 indicated with a dashed line as well as $FSC_{average}$ (blue) and self and cross-validated correlations FSC_{work} (red) and FSC_{test} (green), respectively, shown for (**d**) state I, (**e**) state II, (**f**) state III, (**g**) state IV and (**h**) RF3-70S complex.

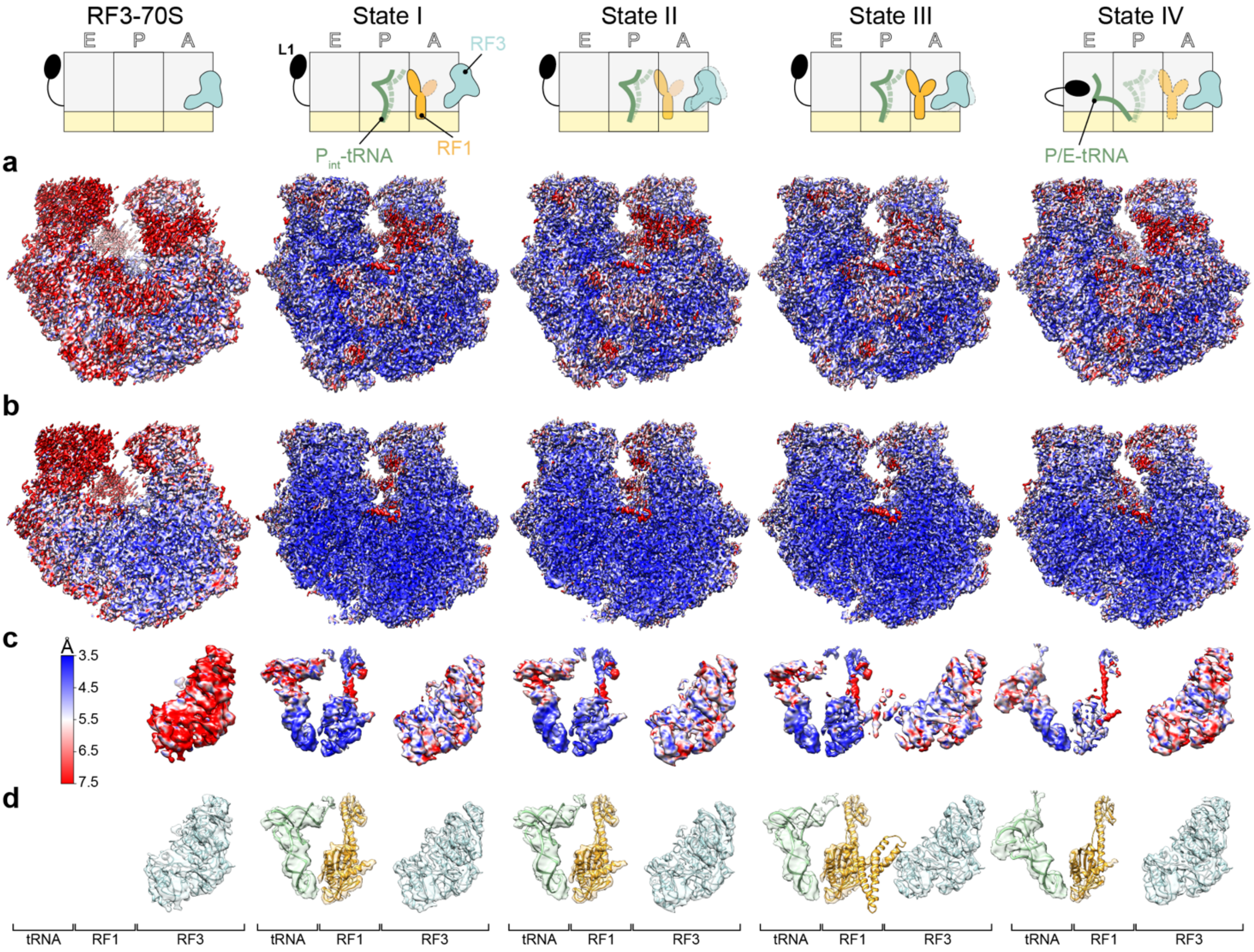

Supplementary Figure 2 Local resolution and molecular models for States I-IV and RF3-70S complex. (**a**) Overview (with schematics shown in first row) and (**b**) transverse section of the cryo-EM maps of the RF3-70S complex and states I-IV colored according to local resolution. Cryo-EM map density for ligands colored according to (**c**) local resolution, or (**d**) with fitted molecular models (displayed as ribbons) and transparent densities for RF3 (cyan), RF1 and tRNA (green).

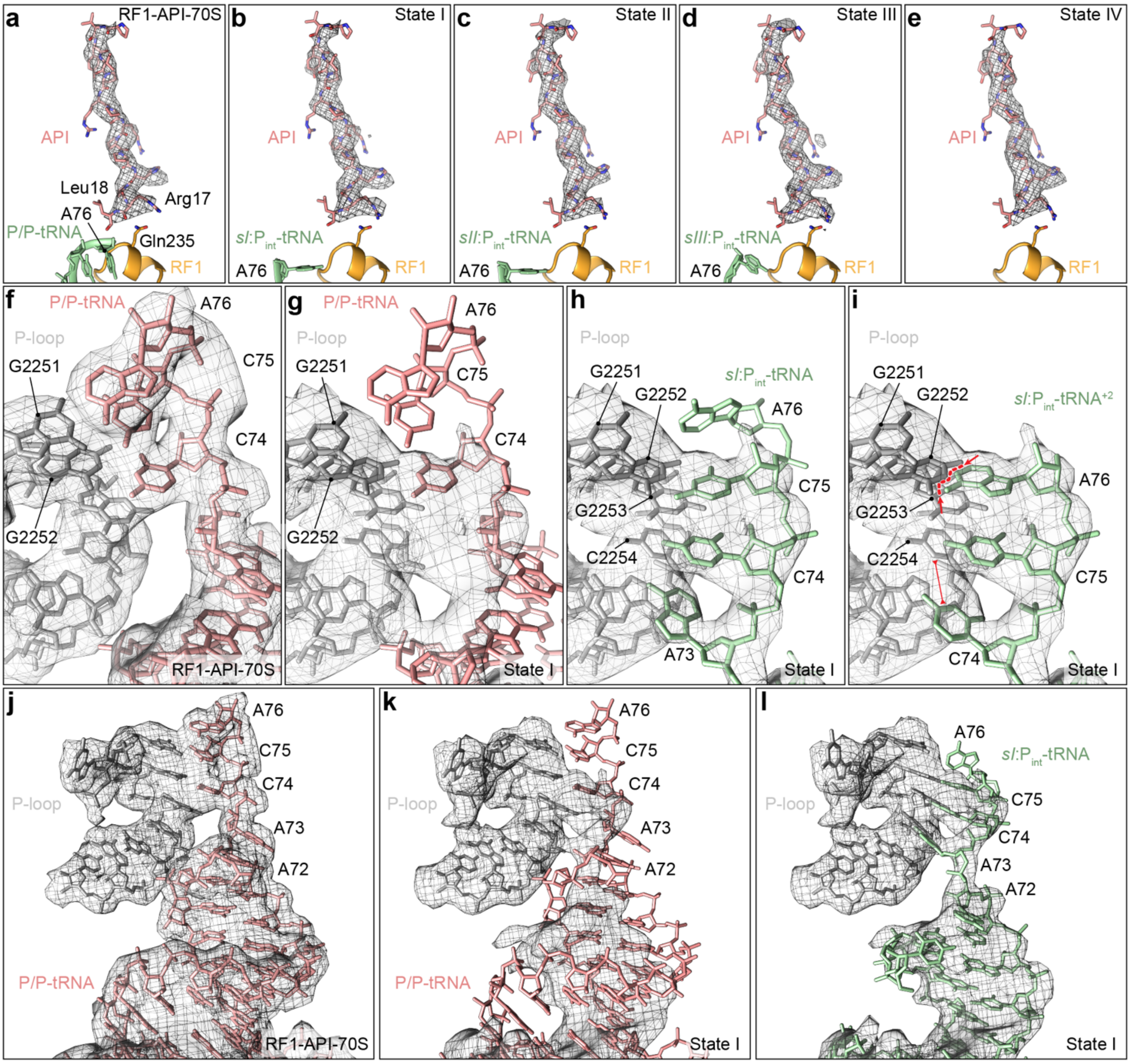
a
RF1-API-70S
b
State I
c
State II
d
State III
e
State IV
API
Leu18
Arg17
A76
P/P-tRNA
Gln235
RF1
sI:Pint-tRNA
sII:Pint-tRNA
sIII:Pint-tRNA
f
P/P-tRNA
A76
P-loop
G2251
C75
C74
G2252
RF1-API-70S
g
State I
h
G2252
sI:Pint-tRNA
G2253
C2254
A73
i
sI:Pint-tRNA+2
j
A72
k
l

Supplementary Figure 3 Interactions at the peptidyltransferase center in states I-IV. (**a-e**) The cryo-EM electron density (grey mesh) for API (salmon) bound within the ribosomal tunnel of (**a**) the RF1-API-70S complex[26] and (**b-e**) states I-IV. The relative position of the (**a**) P/P-site tRNA (green) and (**b-e**) P_{int}-tRNA are shown with A76 of the CCA-end labelled as well as Gln235 of the GGQ motif of RF1. (**f-i**) The cryo-EM electron density (grey mesh) for the (**f**) P/P-site tRNA (salmon) in the RF1-API-70S complex[26] and (**g-i**) the P_{int}-tRNA (green) in state I with relative position of (**g**) canonical P/P-tRNA (salmon), (**h-i**) P_{int}-RNA (green) with (**h**) one or (**i**) two nucleotide shift in the interaction of the CCA-end with the P-loop. Red dashes in (**i**) indicate steric clashes between A76 and G2252, whereas red line indicates suboptimal distance for basepairing between C74 and C2254. (**j-l**) The cryo-EM electron density (grey mesh) for the (**j**) P/P-site tRNA (salmon) in the RF1-API-70S complex[26] and (**k,l**) cryo-EM electron density (grey mesh) for State I with (**k**) superimposition of a canonical P/P-site tRNA (salmon) and (**l**) the P_{int}-tRNA with one nucleotide shift, showing the location of nucleotide A73.

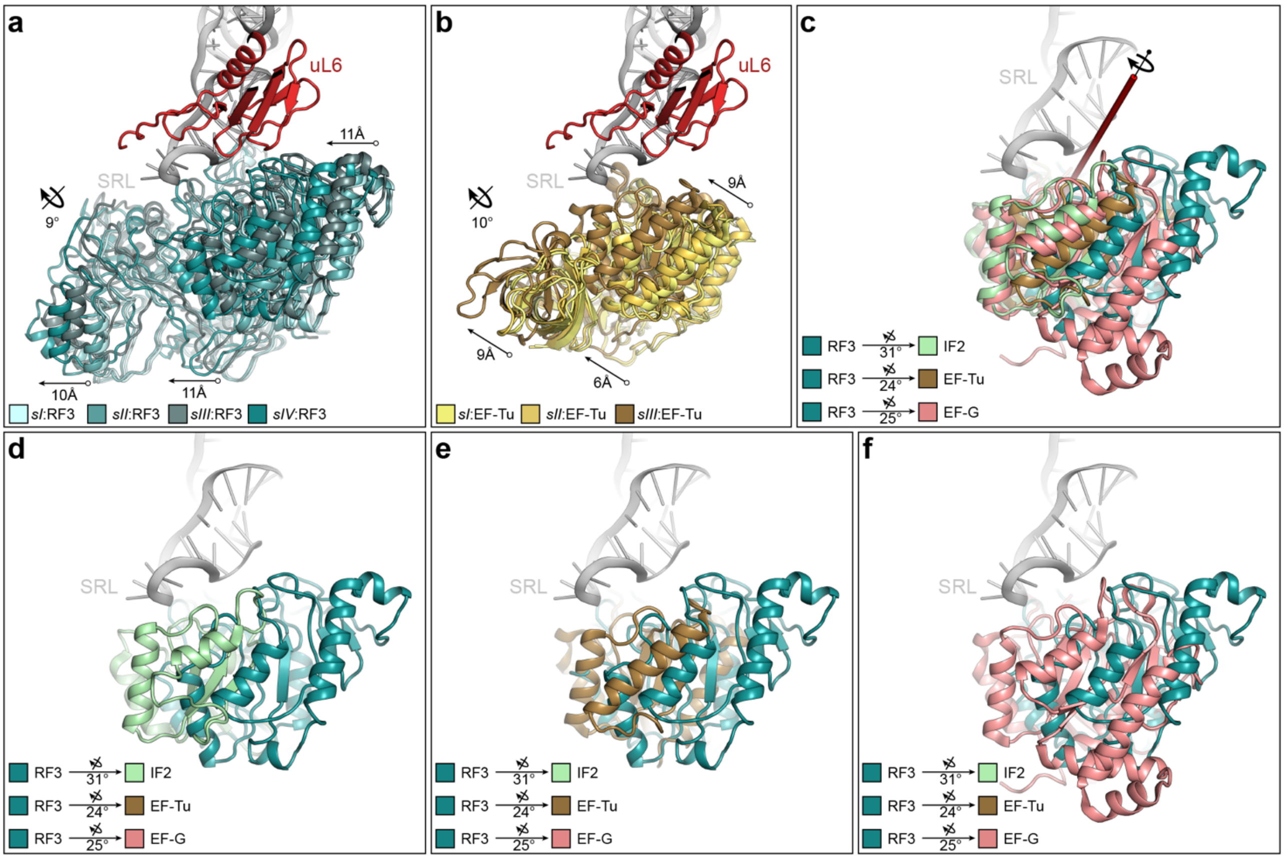

Supplementary Figure 4 Interaction of the G-domain of RF3 with the ribosome. (**a**) Superimposition of RF3 (different shades of green) from states I-IV, relative to the sarcin-ricin loop (SRL, grey) and ribosomal protein uL6 (red) of the LSU. (**b**) Same view as (**a**) but with alignment of three different states of EF-Tu (PDB ID 5UYK; 5UYL; 5UYM)[42]. (**c-f**) Superimposition of RF3 from state IV with (**c**) IF2, EF-Tu and EF-G together, or separately with (**d**) IF2 (lime, PDB ID 3JCN)[40], (**e**) EF-Tu (brown, PDB 5UYM)[42] and (**f**) EF-G (salmon, PDB 3JA1)[74]. In (**c**), the axis of rotation that aligns the G-domains of RF3 to the G-domain of the other translational GTPases is shown.

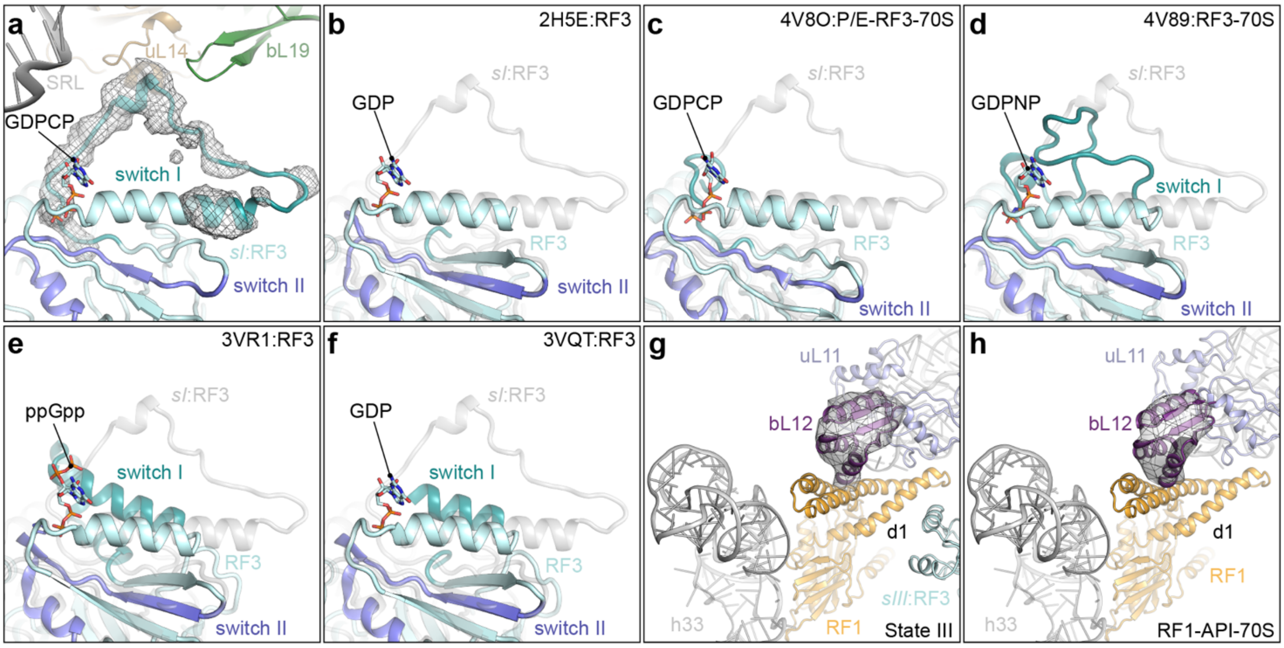

Supplementary Figure 5 Interaction of the switch I loop with large ribosomal subunit**.** (**a**) The ordered switch I loop (teal) of RF3 in state I is shown with the electron density (grey mesh) and relative to the SRL (grey) and ribosomal proteins uL14 (tan) and bL19 (green). (**b-f**) same view as (**a**) but with aligned structures showing disordered switch I loop in (B) RF3-GDP crystal structure (PDB ID 2H5E)[17] and (**c**) RF3-70S complex with P/E-site tRNA (PDB ID 4V8O)[28] and ordered switch I loop (teal) in (**d**) RF3-70S complex without tRNA (PDB ID 4V89)[29], (**e-f**) RF3 crystal structure in complex with (**e**) ppGpp (PDB ID 3VR1)[18] and (**f**) GDP (PDB ID 3VQT)[18]. In (**a-f**), the switch II loop (slate) is shown highlighted and in (**b-f**) the conformation of the switch I loop from (**a**) is shown in transparent grey for reference. (**g-h**) Cryo-EM electron density (grey mesh, filtered to 7 Å) for the CTD of bL12 (purple) interacting with domain 1 (d1) of RF1 (orange) and ribosomal protein uL11 (light blue) in (**g**) state III and (**h**) the RF1-API-70S complex[26].

Supplementary Table 1 Rotational analysis of the 30S ribosomal subunit.

**Complex**	Head swivel (°)	Body/platform rotation (°)
State I	1.1	0.8
State II	1.6	1.7
State III	1.6	5.5
State IV	3.6	9.6
RF3-70S complex	5.7*	8.2
4v6r (A-tRNA + P-tRNA)	4.6	8.8
4v9d (P-tRNA only)	-1.2	1.1
4v9d (P/E-tRNA; RRF)	2.6	9.9
4v89 (RF3)	13.6	9.1
4v8o (P/E-tRNA; RF3)	3.6	10.1
5LZF (P/E-tRNA**)	3.2	10.4

*average head swivel; **fMetSec-$tRNA^{Sec}$
