# Supplementary material for: Visualization of translation termination intermediates trapped by the Apidaecin 137 peptide during RF3-mediated recycling of RF1
Source: Nat Commun. 2018 Aug 3;9:3053. doi: 10.1038/s41467-018-05465-1 (PMC6076264; doi:10.1038/s41467-018-05465-1)
Supplement: Supplementary file 3 — Description of Additional Supplementary Files [file 41467_2018_5465_MOESM3_ESM.pdf]

Descriptions of Additional Supplementary Files

File Name: Supplementary Movie 1
Description: Comparison of movements between state I to IV and RF3-70S. (a-d) The structures of state I-IV and the RF3-70S complex shown with view (a) into the factor binding site, (b) from above with transparent SSU and LSU, and (c-d) interface views of the (c) LSU and (d) SSU. The SSU (yellow), LSU (grey), RF1 (orange), tRNA (green) and RF3 (cyan) are shown as surface representations.

File Name: Supplementary Movie 2
Description: Comparison of RF3-70S complex with RF3-70S crystal structures. (a) The RF3-70S X-ray crystallography structures without tRNA (PDB ID 4V89)29 and with hybrid P/E-site tRNA (PDB ID 4V8O)28. (b-c) The RF3-70S complex compared with the RF3-70S X-ray crystallography structures (b) without tRNA (PDB ID 4V89)29 and (c) with hybrid P/E-site tRNA (PDB ID 4V8O)28.
