# Supplementary figures and images for: Visualization of translation termination intermediates trapped by the Apidaecin 137 peptide during RF3-mediated recycling of RF1
Source: Nat Commun. 2018 Aug 3;9:3053. doi: 10.1038/s41467-018-05465-1 (PMC6076264; doi:10.1038/s41467-018-05465-1)

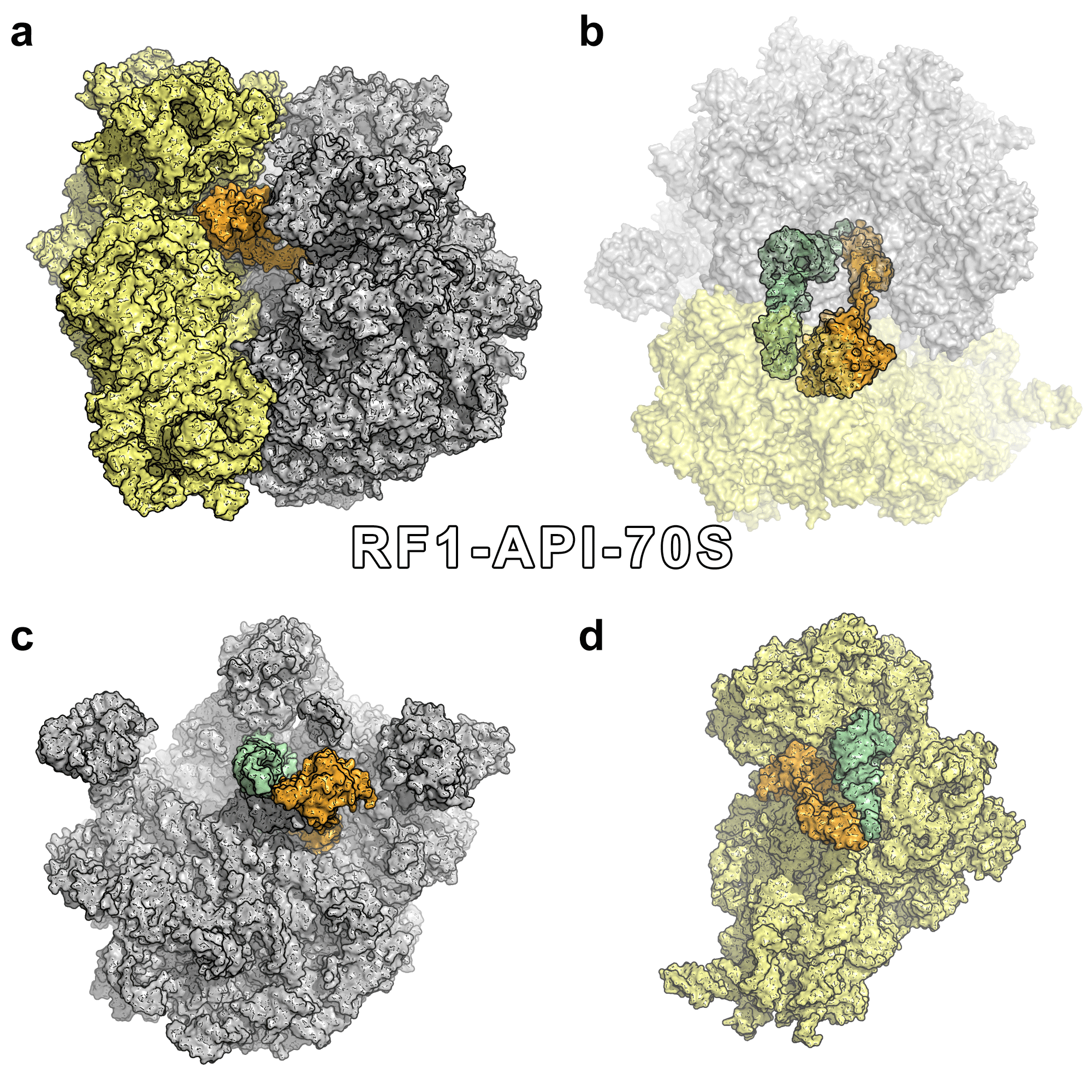

Supplement: Supplementary file 4 — Supplementary Movie 1 [file 41467_2018_5465_MOESM4_ESM.gif]

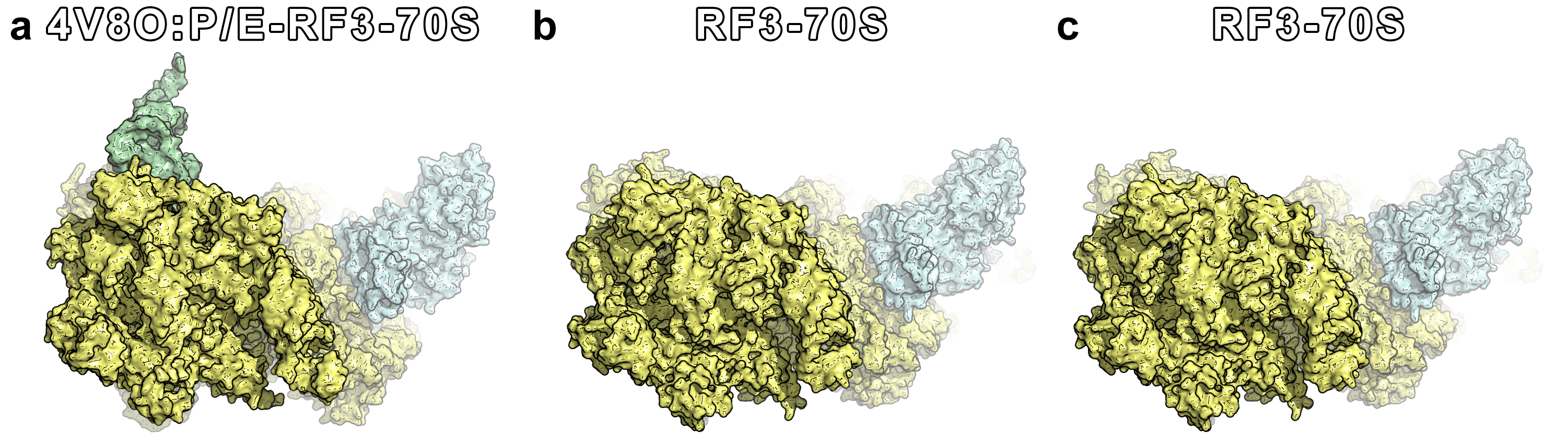

Supplement: Supplementary file 5 — Supplementary Movie 2 [file 41467_2018_5465_MOESM5_ESM.gif]
